# Simultaneous Decontamination for Ammonia Nitrogen and Phosphate Efficiently by Crystal Morphology MgO-Coated Functional Biochar Derived from Sludge and Sunflower Stalk

**DOI:** 10.3390/toxics13070577

**Published:** 2025-07-09

**Authors:** Zhiwei Li, Jingxin Huang, Weizhen Zhang, Hao Yu, Yin Wang

**Affiliations:** 1 State Key Laboratory of Regional and Urban Ecology, Institute of Urban Environment, Chinese Academy of Sciences, Xiamen 361021, China; jxhuang@iue.ac.cn (J.H.); wzzhang@iue.ac.cn (W.Z.); haoyu@iue.ac.cn (H.Y.); 2 State Key Laboratory of Advanced Environmental Technology, Institute of Urban Environment, Chinese Academy of Sciences, Xiamen 361021, China; 3 University of Chinese Academy of Sciences, Beijing 100049, China; 4 Ningbo (Beilun) Zhongke Haixi Industrial Technology Innovation Center, Ningbo 315000, China

**Keywords:** sludge, biochar, co-pyrolysis, modification, decontamination

## Abstract

Eutrophication driven by nitrogen and phosphorus discharge remains a critical global environmental challenge. This study developed a sustainable strategy for synergistic nutrient removal and recovery by fabricating MgO-coated biochar (Mg-MBC600) through co-pyrolysis of municipal sludge and sunflower stalk (300–700 °C). Systematic investigations revealed temperature-dependent adsorption performance, with optimal nutrient removal achieved at 600 °C pyrolysis. The Mg-MBC600 composite exhibited enhanced physicochemical properties, including a specific surface area of 156.08 m^2^/g and pore volume of 0.1829 cm^3^/g, attributable to magnesium-induced structural modifications. Advanced characterization confirmed the homogeneous dispersion of MgO nanoparticles (~50 nm) across carbon matrices, forming active sites for chemisorption via electron-sharing interactions. The maximum adsorption capacities of Mg-MBC600 for nitrogen and phosphorus reached 84.92 mg/L and 182.27 mg/L, respectively. Adsorption kinetics adhered to the pseudo-second-order model, indicating rate-limiting chemical bonding mechanisms. Equilibrium studies demonstrated hybrid monolayer–multilayer adsorption. Solution pH exerted dual-phase control: acidic conditions (pH 3–5) favored phosphate removal through Mg_3_(PO_4_)_2_ precipitation, while neutral–alkaline conditions (pH 7–8) promoted NH_4_^+^ adsorption via MgNH_4_PO_4_ crystallization. XPS analysis verified that MgO-mediated chemical precipitation and surface complexation dominated nutrient immobilization. This approach establishes a circular economy framework by converting waste biomass into multifunctional adsorbents, simultaneously addressing sludge management challenges and enabling eco-friendly wastewater remediation.

## 1. Introduction

Eutrophication is a pressing global environmental concern, as it severely damages aquatic ecosystems and poses threats to drinking water safety and human health [1]. While external factors such as sunlight, temperature, water depth, and flow rate play a role, the accumulation of nitrogen (N) and phosphorus (P) in water is the primary cause of eutrophication [2,3]. Addressing the pollution of nitrogen and phosphorus and mitigating eutrophication are of urgent environmental concern. To tackle this issue, various methods and technologies, including chemical precipitation, membrane separation, electrochemical processes, and adsorption, have been employed based on the distinct forms and transformation pathways of nitrogen and phosphorus in wastewater [4]. Among these, adsorption technology has emerged as a predominant method for treating wastewater containing nitrogen and phosphorus, given its broad applicability, straightforward operational process, consistent performance, and absence of secondary pollution [5]. Nevertheless, the significant cost of adsorption materials hinders its widespread adoption. Biochar, a carbonaceous solid produced through the high-temperature pyrolysis of biomass in an oxygen-limited environment, has gained attention as a potential adsorbent due to its affordability and innovative nature [6,7].

With the sustained and rapid growth of China’s economy, the capacity of urban wastewater treatment facilities has expanded substantially, leading to a proportional increase in sludge generation as an inevitable by-product of wastewater treatment processes. The sludge output from urban and county sewage treatment plants nationwide exceeded 71.145 million tons (based on a moisture content of 80%) in 2023, highlighting a consistent annual growth trend [8]. Such an increase poses a formidable challenge for the expedient, safe, and large-scale treatment of sludge [9]. Compared with conventional sludge disposal options such as landfilling, composting, and incineration, sludge pyrolysis provides a potential technology for harmless, reduced, and resource-based treatment that results in minimal heavy metal leaching into the final residue and also produces valuable by-products in the form of sludge-based biochar, bio-oil, and biogas [10]. Sludge-based biochar has adsorption properties and can be modified to produce adsorbents to improve sewage treatment processes, such as the adsorption of refractory organic matter or phosphorus to support intra-plant recycling [11]. Moreover, it can be used as a cheaper alternative to activated-carbon adsorbents to purify industrial wastewater, owing to its excellent adsorption properties.

The production of sludge-based biochar through pyrolysis offers the advantages of readily available raw materials and a high density of the end product, positioning it as an effective solution for sludge disposal and management [12]. However, the limited fixed carbon content in sludge results in biochar with a reduced specific surface area and less developed pore structure. In contrast, cellulose-rich biomass, such as agricultural and forestry wastes, has a higher carbon content compared to sludge [10]. As a result, biochar derived from this cellulose-rich biomass exhibits superior specific surface area and porosity. Nevertheless, its lower density makes storage and transportation of biochar from lignocellulosic biomass challenging.

Importantly, the metal elements found in sludge, including calcium, magnesium, aluminum, and iron, play a crucial role in pollutant removal. Thus, co-pyrolysis of sludge with lignocellulosic biomass can mitigate the individual limitations of each method, offering comprehensive benefits for the practical use of the co-derived biochar. The addition of organic solid waste to sludge enhances the carbon content and porous structure of the resulting biochar [13]. Consequently, biochar from co-pyrolysis of sludge and lignocellulosic biomass has broader application prospects [14]. Co-pyrolysis of these materials exhibits a notable synergistic effect, leading to exceptionally high specific and micropore surface areas [15]. Existing research indicates that both composite and core–shell biochars possess a larger specific surface area compared to sludge-based biochar [16]. Moreover, studies have confirmed that the specific surface area of an adsorbent significantly influences the adsorption of NH_4_^+^. Beyond the specific surface area, the type and quantity of surface functional groups also play pivotal roles [17].

Sludge-based biochar serves as a potent adsorbent for the removal of pollutants from sewage. However, its native form exhibits a limited adsorption capacity for nitrogen and phosphorus [18]. To enhance its performance, modifications to the sludge-based biochar are imperative. The primary techniques for such modifications encompass chemical, physical, and physicochemical approaches, including oxidation, reduction, and metal loading [19]. Among these, metal loading emerges as a particularly straightforward and effective method [20]. This is because the introduction of metal sites can counterbalance the inherent negative charge on the solid surface, thereby diminishing repulsive forces between the biochar and the adsorbate. Moreover, the strong affinity between metal active sites and phosphates ensures a high adsorption capacity. A uniform distribution of metal sites on the biochar surface further optimizes its utilization efficiency by facilitating even distribution within its porous matrix. Notably, compared to unmodified biochar, magnesium-enhanced biochar possesses a greater number of surface functional groups [21]. Furthermore, the magnesium in the biochar interacts with various pollutants in water, underscoring the efficacy of Mg-modified biochar as an adsorbent. Such biochar also holds promise as a soil conditioner, enhancing both soil fertility and its capacity for water retention [22,23]. Given that plants require essential elements like nitrogen and phosphorus, post-adsorption biochar can serve as a slow-release fertilizer, promoting plant growth [24]. Additionally, given the role of magnesium in chlorophyll synthesis, Mg-modified biochar presents itself as a promising adsorbent for the extraction of nitrogen and phosphorus from water [25]. When used for soil enhancement, this adsorbed Mg-modified biochar offers added benefits [25].

However, there are currently few studies about the mechanistic aspects of the simultaneous synergistic removal of nitrogen and phosphorus by preparing functional biochar from sludge and biomass before pyrolysis modification. Therefore, a comprehensive understanding of this synergistic effect, along with the associated mechanisms, remains elusive.

This study explores the collaborative creation of magnesium-enhanced biochar and its nitrogen–phosphorus adsorption–recovery mechanisms. We employed a magnesium-centric modification strategy combined with co-pyrolysis technology to effectively convert municipal sludge and sunflower stalk into functional biochar materials with superior nitrogen- and phosphorus-removal capabilities. Through a systematic investigation of the regulatory mechanisms of magnesium salt pretreatment parameters and co-pyrolysis conditions on biochar’s physicochemical properties, we elucidated the interfacial migration pathways of nitrogen–phosphorus pollutants in multiphase systems and revealed the evolution mechanisms of adsorption sites, along with the nutrient-recovery potential, in magnesium-modified biochar. These findings provide a theoretical basis and technical framework for the valorization of sludge–biomass waste and the restoration of aquatic ecosystems.

## 2. Experimental Materials and Methods

### 2.1. Raw Material Pretreatment and Co-Pyrolysis Method

The sludge used in the experiment was municipal sludge with a moisture content of about 80% obtained from a sewage treatment plant in Xiamen. After being retrieved, it was dried in an oven at 105 °C to constant weight. Sunflower straw was sourced from the market, washed with water to remove impurities, and then dried in an oven at 85 °C to constant weight. The dried sludge and sunflower straw were crushed to 40–60 mesh for later use. Magnesium-loaded biomass was prepared by using mixed raw materials to immerse in a magnesium-containing solution that simulates seawater. The specific process is as follows: After the sludge and sunflower straw are mixed in a mass ratio of 1:1, 5 g is weighed and added to 50 mL of MgSO_4_ solution (wherein the magnesium concentration is 1.54 g/L, which is consistent with the average magnesium concentration in seawater), then shaken for 2 h, and soaked at room temperature for 22 h. The the solid–liquid mixture was separated via filtration, yielding solid residue and aqueous phase, and the resulting solid was dried at 85 °C to constant weight, and a powder sample less than 120 mesh was collected by sieving for later use. The pyrolysis experiment was carried out in a homemade pyrolysis carbonization experimental device similar to recent research [26]. Specific experimental method: First, 20.0 g of sample (the dry-weight ratio of sludge to sunflower straw was 1:1) was placed in a quartz tube and purged with argon (99.99% purity) for 60 min, and then the temperature was increased by adjusting the flow rate to 100 mL/min of argon. The pyrolysis temperature was increased from room temperature to the target temperature at a rate of 10 °C/min^−1^, and the target temperature (300–700 °C) was maintained for 60 min. For simplicity, SBC stands for sewage sludge biochar, MBC stands for co-pyrolysis biochar of sewage sludge and sunflower stalk, and Mg-MBC stands for magnesium-loaded modified co-pyrolysis biochar. The biochar was named Mg-MBCX according to the pyrolysis temperature, X; for example, the biochar prepared at 600 °C was named Mg-MBC600. All experiments were performed in triplicate. All pyrolysis experiments were repeated three times, and the repeated samples obtained from each experiment were mixed evenly. The finely sieved (<1 mm) biochar was extracted three times with deionized water, dried at 85 °C for 24 h, and sealed and stored in a desiccator for further analysis and testing.

### 2.2. Analysis and Characterization of Material

To investigate the pyrolysis characteristics of the raw material samples, thermogravimetric (TG) analysis was performed using a TG analyzer (Netzsch TG 209, Selb, Germany). The loading amount was about 10 mg. The raw material samples were heated from room temperature to 950 °C at a heating rate of 10 °C/min, under a nitrogen flow. The carrier gas was high-purity nitrogen with a gas flow rate of 60 mL/min. The contents of C, H, and N in the sample were determined by using an elemental analyzer (Vario MAX, Hanau, Germany). The pH of the samples (sample: deionized water = 1:20, *w*/*v*) was measured for the suspension of the mixture of biochar and water by a digital pH meter (UB-7, Denver, CO, USA). The magnesium content was analyzed using inductively coupled plasma optical emission spectrometry (ICP-OES, Optima 7000DV, Berkeley, CA, USA). The N_2_ adsorption–desorption isotherms were measured at −196.15 °C using a Micromeritics surface analyzer (ASAP 2020M+C, Houston, TX, USA) to calculate the specific surface area of the samples. The surface morphology of the samples was characterized by a scanning electron microscope (SEM, S-4800, Marunouchi, Japan). Elemental composition and mapping were analyzed by energy-dispersive X-ray spectroscopy (EDS, Hitachi, Marunouchi, Japan). X-ray diffraction (XRD) with Cu Kα radiation (40 kV, 40 mA) was used in a 2θ range of 5° to 90°, using a diffractometer (X’Pert Pro, Almelo, The Netherlands) to confirm the crystalline phases in the raw material and biochars. The composition of phosphorus in the modified biochar before and after adsorption was studied by X-ray photoelectron spectroscopy (XPS), using an Al Kα X-ray source (Axis Supra, Manchester, UK).

### 2.3. Adsorption Experiment

In the adsorption experiments, the effects of different initial concentrations, contact time, pH value, and temperature were mainly studied. To evaluate adsorption performance across varying initial concentrations, 0.1 g of adsorbent was added to 50 mL of ammonia nitrogen (NH_4_^+^-N) and phosphate (PO_4_^3−^-P) solutions, with concentrations ranging from 5 to 100 mg/L (initial pH uniformly adjusted to 6.25). The mixture was stirred at 150 rpm in a temperature-controlled shaker for 24 h to ensure equilibrium. After the flask was removed from the shaker, the suspension was filtered through a 0.45 μm filter membrane. The concentration of nitrogen and phosphorus in the suspension was analyzed using Nessler’s reagent colorimetry at a wavelength of 420 nm and a molybdate blue spectrophotometer at a wavelength of 700 nm, respectively. In the adsorption experiment, the adsorption capacity was calculated as follows:

q_e_ = ((C_0_ − C_e_)V)/m (1)

where C_0_ (mg/L) and Ce (mg/L) are the concentrations of the initial solution and the equilibrium solution, respectively; V (L) is the volume of the solution; and m (g) is the mass of the sample added to the solution.

Adsorption kinetics were established at different shaking times. The specific experimental steps were as follows: 0.1 g of adsorbent sample was added to 50 mL of 20 mg/L NH_4_Cl/KH_2_PO_4_ solution in a 100 mL flask and stirred at 150 rpm in a thermostatic shaker at room temperature (25 °C). The supernatant was sampled at a series of time points: 0, 10, 30, 60, 120, 200, and 480 min; the measurements were repeated three times. The effect of pH on the adsorption capacity was studied in the pH range of 2–10. HCl and NaOH (0.1 mol/L) were used to adjust the pH of the solution. A total of 0.1 g of adsorbent was added to 50 mL of 20 mg/L NH_4_Cl/KH_2_PO_4_ solution in a 100 mL flask and stirred at 150 rpm for 24 h, at room temperature (25 °C), in a thermostatic shaker. Each set of experiments was repeated three times. To determine the effect of temperature on nitrogen and phosphorus adsorption, adsorption was performed at 25, 35, and 45 °C, following a similar procedure as the adsorption isotherm. All experiments were performed in triplicate.

### 2.4. Statistical Analysis

The statistical significance of differences among different biochars was conducted through analysis of variance (ANOVA). The level of accepted statistical significance was *p* < 0.05.

## 3. Results and Discussion

### 3.1. Analysis of Basic Physicochemical Properties of Raw Materials

The results of the industrial analysis (refer to Table 1) demonstrated marked disparities between biomass and sludge. Most notably, the ash content in sludge was substantially higher compared to biomass, while biomass contained more than double the volatile matter compared to sludge. Moreover, the moisture content in sludge post-drying was relatively low. Sewage sludge exhibits significantly higher inherent ash content (63.74 wt%) and nitrogen concentration (1.62 wt%) compared to sunflower stalk (3.55 wt% ash; 0.29 wt% nitrogen). Conversely, sunflower stalk demonstrates superior fixed carbon (6.24 wt%), elemental carbon content (47.04 wt%), and BET surface area (26.87 m^2^/g). The elevated ash and nitrogen fractions in sludge may reduce its energy density and increase nitrogenous emissions during thermochemical processing. In contrast, the compositional profile of sunflower stalk renders it more suitable for energy-recovery applications, particularly through pyrolysis.

**Figure 1:**
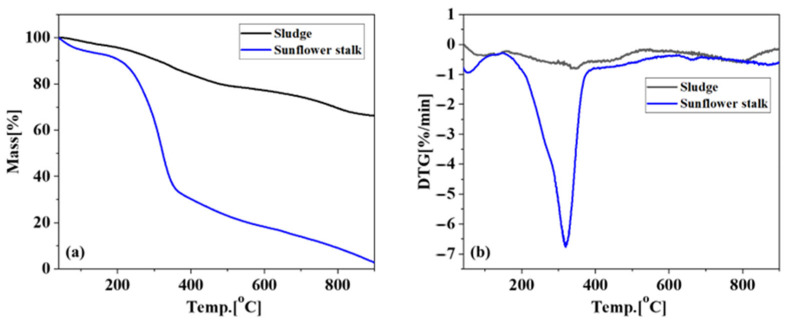
Thermogravimetric curves of sludge and sunflower stalks: (**a**) TG and (**b**) DTG.

Figure 1 presents the thermogravimetric (TG) and derivative thermogravimetric (DTG) profiles of sludge and sunflower stalk samples under nitrogen atmosphere. The sunflower stalk pyrolysis was characterized by a prominent mass loss stage between 260 and 410 °C, exhibiting 56.8% weight reduction. Initial mass loss below 150 °C was attributed to moisture evaporation, while the subsequent asymmetric DTG peak within 260–410 °C corresponded to combined decomposition of cellulose (maximum decomposition at ~350 °C) and gradual lignin degradation (240–540 °C), collectively accounting for 35.63% of total mass loss. Both materials demonstrated accelerated decomposition rates within 320–680 °C. Based on these thermal degradation patterns, the pyrolysis temperature range for subsequent experiments was systematically established as 300–700 °C, with 100 °C increments.

### 3.2. Effect of Preparation Conditions of Magnesium-Modified Biochar on Nitrogen and Phosphorus Adsorption Capacity

**Figure 2:**
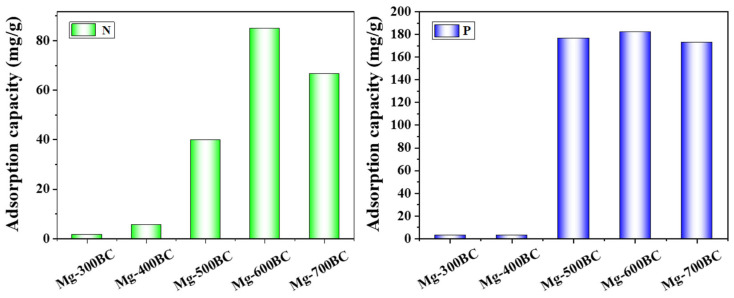
Adsorption capacity of magnesium-loaded biochar prepared at different pyrolysis temperatures for nitrogen and phosphorus.

The effect of different temperatures on the preparation of Mg-modified biochar by co-pyrolysis of sludge and sunflower stalks was investigated, and the biochar prepared at different pyrolysis temperatures was used for the adsorption and recovery of nitrogen and phosphorus in a mixed system of NH_4_^+^ and H_2_PO_4_^−^. As shown in Figure 2, the adsorption of nitrogen and phosphorus by biochar showed a trend of rapid increase and then decrease. The nitrogen and phosphorus adsorption capacity of biochar prepared at 300 °C was only 1.86 mg/g and 3.47 mg/g. When the pyrolysis temperature increased to 500 °C, the nitrogen and phosphorus adsorption capacity of biochar increased to 39.89 mg/g and 176.78 mg/g. When the pyrolysis temperature increased to 600 °C, the nitrogen and phosphorus adsorption capacity of biochar increased to 84.92 mg/g and 182.27 mg/g. However, when the pyrolysis temperature was further increased to 700 °C, the nitrogen and phosphorus adsorption capacity of biochar decreased compared with the biochar prepared at 600 °C, which were 66.49 mg/g and 172.95 mg/g, respectively. This may be due to the fact that with the increase in pyrolysis temperature, the organic matter in the sludge and sunflower stalks gradually decomposed and escaped with the increase in temperature. When the temperature was further increased, the carbon content in the solid phase decreased, some micropores were destroyed, and the specific surface area decreased [13]. At the same time, the morphology of magnesium loaded on the carbon also changed with the influence of pyrolysis temperature [27]. According to the above results, the subsequent pyrolysis temperature was determined to be 600 °C.

**Figure 3:**
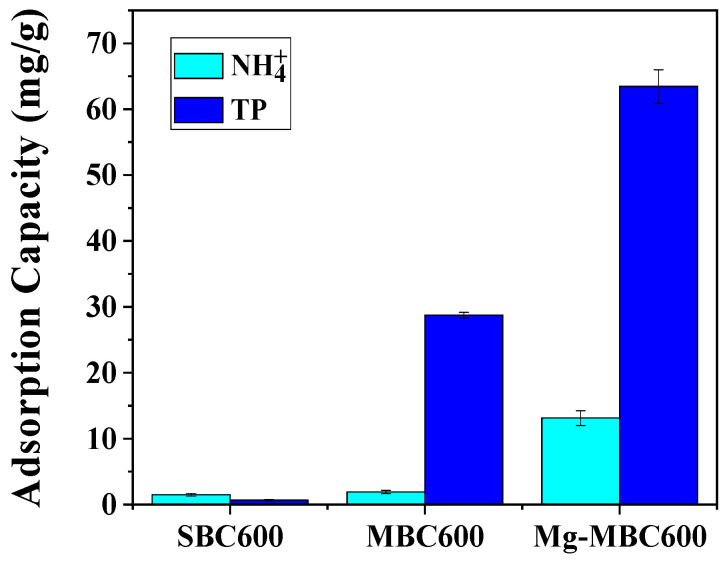
Nitrogen and phosphorus adsorption capacity of biochar before and after modification.

Figure 3 reveals distinct adsorption performance variations among sludge-based biochar (SBC600), co-pyrolyzed sludge–biomass biochar (MBC600), and magnesium-modified biochar for ammonia nitrogen (NH_4_^+^-N) and total phosphorus (TP). SBC600 exhibited the lowest NH_4_^+^-N adsorption capacity (1.48 mg/g), while MBC600 showed a marginal improvement (1.90 mg/g). Magnesium modification significantly enhanced NH_4_^+^-N adsorption to 13.12 mg/g. This progression is mechanistically explained by (1) the enhanced surface area of co-pyrolyzed biochar, which facilitates physical adsorption; and (2) magnesium-induced chemisorption through struvite (MgNH_4_PO_4_·6H_2_O) precipitation under co-existing NH_4_^+^-N and phosphorus conditions, as documented in prior studies. For TP removal, SBC600 exhibited constrained adsorption capacity (0.65 mg/g) due to phosphate dissolution–leaching equilibria overriding adsorption contributions. While SBC600 contains calcium and iron, these metals exist as thermodynamically stable phosphates (FePO_4_, Ksp = 1.3 × 10^−22^; Ksp Ca_3_(PO_4_)_2_ = 2.0 × 10^−29^) that occupy structural coordination sites (XPS O 1s: 531.2 eV), precluding available adsorption centers. Co-pyrolysis with sunflower stalks (1 wt% Ca) increased TP adsorption to 28.76 mg/g, attributed to calcium-mediated binding mechanisms. Magnesium modification achieved optimal TP adsorption (63.43 mg/g), where MgO deposition on biochar surfaces/pores provided abundant phosphate-binding sites. Concurrently, magnesium ammonium phosphate precipitation was promoted in NH_4_^+^-N-rich environments, synergistically enhancing TP removal [28].

Distinct variations in elemental composition and structural characteristics among biochar samples are delineated by Table 2. Sludge-derived biochar (SBC) was characterized by low carbon content (10.63 wt%) and moderate surface properties (SBET, 49.62 m^2^/g; pore volume, 0.1685 cm^3^/g; and average pore diameter, 14.87 nm). Co-pyrolysis of sludge with biomass substantially elevated carbon content to 46.57 wt% while refining pore structure (SBET, 124.36 m^2^/g; pore volume, 0.1764 cm^3^/g; and pore diameter, 4.23 nm). Magnesium-modified co-pyrolysis (Mg-SBC600) achieved optimal textural parameters (SBET, 156.08 m^2^/g; and pore volume, 0.1829 cm^3^/g), representing a 214% surface-area enhancement compared to SBC. All biochars exhibited mesoporous structures (pore diameters > 1 nm), though Mg-MBC600 demonstrated superior microstructural refinement through reduced average pore diameter (4.23 nm vs. 14.87 nm for SBC). Notably, sludge-based biochar production yielded inferior structural metrics compared to co-pyrolyzed counterparts, particularly in surface area (49.62 vs. 124.36–156.08 m^2^/g) and pore volume (0.1685 vs. 0.1764–0.1829 cm^3^/g). The synergistic effects of magnesium incorporation and biomass co-pyrolysis were thus identified as critical factors for developing high-surface-area adsorbents with enhanced porosity [29].

**Figure 4:**
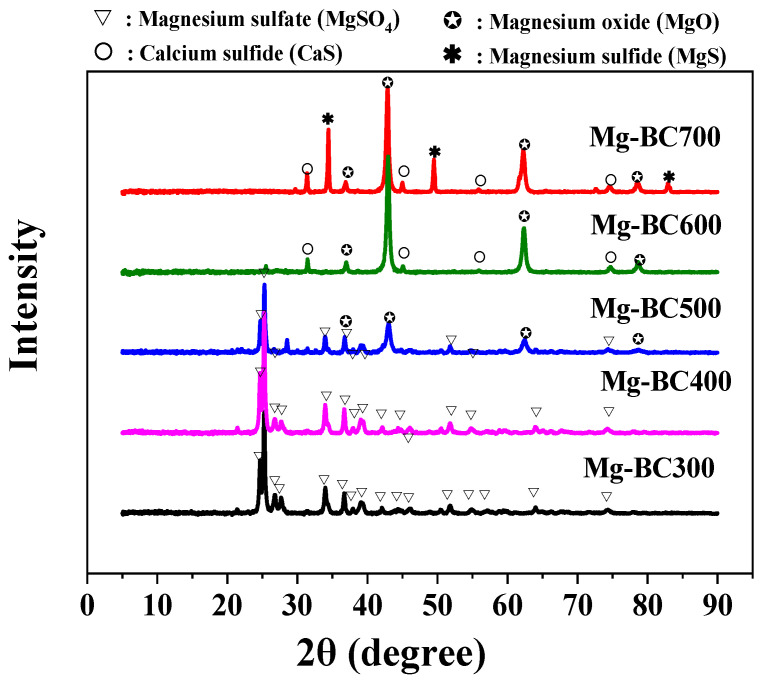
XRD spectra of magnesium-loaded biochar from co-pyrolysis for sludge and sunflower stalk prepared under different pyrolysis temperatures.

Figure 4 presents the X-ray diffraction (XRD) spectra of magnesium-loaded co-pyrolyzed biochar synthesized under varying pyrolysis temperatures. Distinct diffraction peaks were observed in biochar pyrolyzed at 600 °C and 700 °C, corresponding to angular positions of 37.12°, 43.13°, 62.53°, and 78.62°, which are attributed to the (111), (200), (220), and (222) crystallographic planes of magnesium oxide (MgO), respectively. This confirms the crystallization of MgO during high-temperature pyrolysis. Additionally, magnesium sulfide (MgS) and calcium sulfide (CaS) phases were identified in the 700 °C sample, as evidenced by secondary peaks at 53.8° and 65.2°. In contrast, biochar produced at lower temperatures (300 °C and 400 °C) exhibited dominant diffraction features at 24.62°, 25.22°, 33.94°, 34.39°, 36.66°, and 39.03°, indicative of magnesium sulfate (MgSO_4_) surface adsorption. The 500 °C sample demonstrated a transitional phase composition, with coexisting MgSO_4_ and MgO diffraction patterns, suggesting partial thermal decomposition of MgSO_4_ to MgO initiates at this temperature threshold [30].

The synthesis path of Mg-MBC600 is as follows: the mixed raw material of sludge and sunflower stalk biomass is immersed in MgSO_4_ in an aqueous solution, and loaded in the raw material and pores through adsorption. The magnesium-loaded biomass then undergoes a pyrolysis process. During the pyrolysis process, the organic matter (cellulose, protein, etc.) in the biomass decomposes and volatilizes, and the magnesium-containing compounds loaded therein undergo a series of reactions to form MgO/MgS particles. The calcium originally contained in the sunflower stalks and sludge will also participate in the reaction to generate CaS loaded on the biochar. The specific reaction is as follows:

(2) $$\mathrm{MgS}O_{4}\to \mathrm{MgO}+SO_{3}↑$$

(3) $$Mg^{2+}+SO_{4}^{2-}+C\to \mathrm{MgS}+CO_{2}↑$$

(4) $$Ca^{2+}+SO_{4}^{2-}+C\to \mathrm{CaS}+CO_{2}↑$$

**Figure 5:**
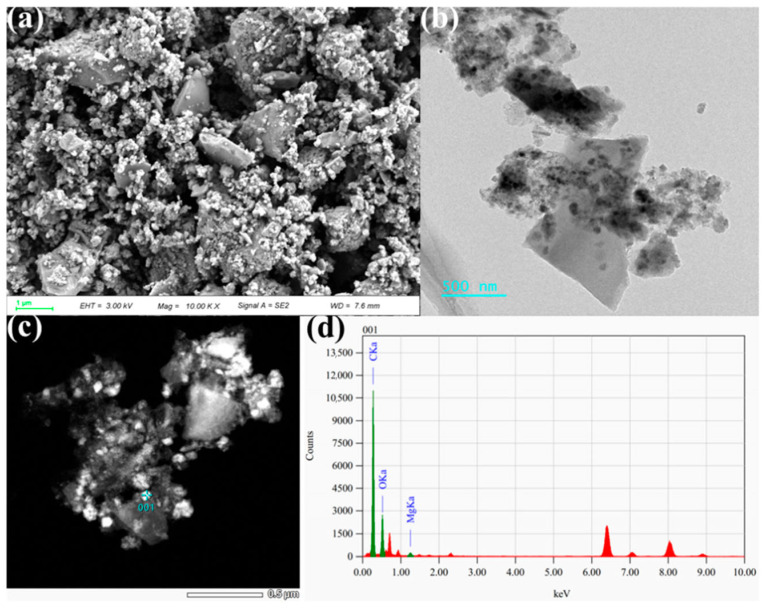
Morphology of Mg-MBC600 and characterization of loaded particles (SEM (**a**), TEM (**b**), EDS (**c**,**d**)).

Figure 5 presents the morphological and compositional characteristics of magnesium-modified co-pyrolyzed biochar, as revealed by scanning electron microscopy (SEM) and transmission electron microscopy (TEM). SEM imaging (Figure 5a) demonstrates that the modified biochar possesses a hierarchically porous architecture, comprising lamellar carbon structures with abundant submicron particulates anchored on their surfaces. Complementary TEM analysis (Figure 5b) reveals uniform dispersion of spherical nanoparticles (~50 nm diameter) across the carbon matrix. Energy-dispersive X-ray spectroscopy (EDS) point analyses (Figure 5c,d) confirm that these surface-bound nanoparticles are magnesium oxide (MgO) crystallites.

### 3.3. Factors and Mechanisms Affecting Adsorption

**Figure 6:**
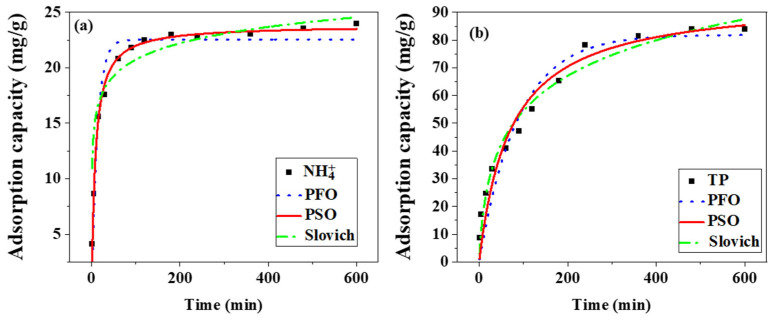
Fitting of nitrogen (**a**) and phosphorus (**b**) adsorption kinetic model of magnesium-modified biochar.

Many models have been developed to model the kinetics of nitrogen and phosphorus adsorption and are widely used to describe the extent and rate of phosphorus adsorption [31]. After modification, the phosphorus adsorption capacity of Mg-loaded co-pyrolysis biochar was significantly improved. Figure 6 delineates the adsorption kinetics of phosphorus onto magnesium-modified co-pyrolyzed biochar, which exhibits enhanced adsorption capacity compared to conventional biochars. The temporal adsorption profile is characterized by a biphasic mechanism: (1) a rapid adsorption phase (0–60 min) dominated by surface chemisorption, followed by (2) a gradual equilibration phase (60–180 min) governed by intraparticle diffusion. Adsorption equilibrium was achieved within 180 min, a timeframe significantly shorter than reported values for unmodified biochars, justifying its selection as the standardized duration for subsequent thermodynamic analyses.

In order to further reveal the adsorption mechanism of ammonia nitrogen and total phosphorus on magnesium-loaded modified co-pyrolysis biochar, the kinetics of nitrogen and phosphorus adsorption on magnesium-loaded modified co-pyrolysis biochar were fitted using pseudo-first-order kinetics, pseudo-second-order kinetics, and the Elovich model. The equations of the three fitting models are as follows:

(5) $$\mathrm{PFO}:\ln\left(q_{2}-q_{t}\right)=\ln q_{e}-{\;k}_{1}t$$

(6) $$\mathrm{PSO}:\;\frac{t}{q_{t}}=\frac{1}{k_{2}q_{e}^{2}}+\frac{t}{q_{e}}$$

(7) $$\mathrm{Elovich}:\;q_{t}=\frac{1}{\beta }\ln(1+\alpha \beta t)$$

where t is the adsorption time; q_e_ (mg/g) is the adsorption amount at equilibrium; q_t_ (mg/g) is the adsorption amount at time t (min); k_1_ (min^−1^), k_2_ (g/mg/min), α (mg/g min), and β (mg/g) are the adsorption rate constants of the pseudo-first-order, pseudo-second-order, and Elovich models.

The kinetic fitting parameters for nitrogen and phosphorus adsorption on magnesium-modified co-pyrolyzed biochar are summarized in Table 3. The correlation coefficients (R^2^) for ammonia nitrogen adsorption were determined as 0.9469 (pseudo-first order), 0.9901 (pseudo-second order), and 0.7956 (intraparticle diffusion), while those for total phosphorus adsorption corresponded to 0.9224, 0.9844, and 0.9716, respectively. The pseudo-second-order kinetic model exhibited superior correlation coefficients (R^2^ = 0.9901 for NH_4_^+^-N; R^2^ = 0.9844 for TP), indicating that the adsorption mechanism was predominantly governed by chemisorption involving electron sharing between MgO active sites and target ions. Three sequential adsorption stages were identified: liquid film diffusion (rapid migration of ions to the biochar surface), surface adsorption as the rate-limiting step (chemisorption at MgO binding sites), intra-particle pore diffusion (slower transport into mesoporous structures). The dominance of surface adsorption was further corroborated by the limited capacity enhancement (<8%) observed after 60 min, suggesting pore diffusion contributes minimally to overall adsorption efficacy. This mechanistic interpretation aligns with the hierarchical porosity (4.23 nm average pore diameter) and MgO surface coverage documented in preceding analyses.

The research related to adsorption isotherms is mainly aimed at the distribution of adsorbates on the adsorbent surface during the adsorption process and the maximum adsorption capacity of pollutants. Isotherm models mainly include Langmuir, Freundlich, and Langmuir–Freundlich models, as shown below:

(8) $${\mathrm{Langmuir}\;\mathrm{model}:\;q}_{e}=\frac{K_{L}q_{m}C_{e}}{1+K_{L}C_{e}}$$

(9) $${\mathrm{Freundlich}\;\mathrm{model}:\;q}_{e}=K_{F}C_{e}^{1/n}$$

(10) Langmuir–Freundlich: qe=kqmCe1n1+kCe1n

where q_e_ (mg/g) is the equilibrium adsorption capacity, C_e_ (mg/L) is the equilibrium concentration, q_m_ (mg/g) is the maximum adsorption capacity, K_L_ (L/mg) is the Langmuir constant, K_F_ [(mg/g)(mg/L)^−n^], and n is the Freundlich constant.

**Figure 7:**
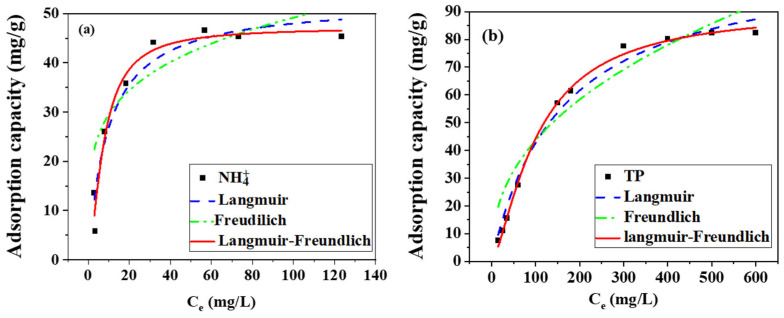
Model fitting of nitrogen (**a**) and phosphorus (**b**) adsorption isotherm on magnesium-modified biochar.

The adsorption behavior was analyzed using three classical isotherm models: the Langmuir model, principally employed to characterize monolayer adsorption on homogeneous surfaces with negligible intermolecular interactions between adsorbed species; the Freundlich model, applied to describe multilayer adsorption on heterogeneous surfaces exhibiting variable adsorption energies; and the Langmuir–Freundlich hybrid model, an empirical formulation accounting for combined monolayer and heterogeneous adsorption mechanisms [32]. This tiered analytical framework enabled systematic discrimination between chemisorption-dominated processes (Langmuir-type) at specific MgO binding sites and physisorption contributions (Freundlich-type) arising from the biochar’s hierarchical porosity, while the hybrid model quantified synergistic interactions between these concurrent adsorption pathways. The adsorption isotherms of magnesium-loaded modified co-pyrolysis biochar for nitrogen and phosphorus are shown in Figure 7, and the relevant fitting parameters are shown in Table 4.

The adsorption characteristics of nitrogen and phosphorus onto magnesium-loaded co-pyrolysis modified biochar were systematically investigated through isothermal analysis. The experimental data demonstrated superior conformity with the Langmuir–Freundlich model, as evidenced by determination coefficients (R^2^) of 0.9567 and 0.9971 for nitrogen and phosphorus adsorption, respectively. This dual-model adherence suggests a hybrid adsorption mechanism involving both monolayer coverage and multilayer heterogeneous adsorption processes. The maximum adsorption capacities were quantitatively determined as 47.17 mg/g for ammonia nitrogen and 91.09 mg/g for total phosphorus, indicating significant nutrient removal potential. Critical analysis of the Langmuir–Freundlich parameter (n) revealed values exceeding 0.5 for both contaminants, which mechanistically corresponds to strong adsorbent–adsorbate interactions. This observation was attributed to the enhanced surface properties imparted by magnesium loading during the co-pyrolysis modification process. The experimental results demonstrate that magnesium-loaded co-pyrolysis modification optimizes biochar’s adsorption performance through three synergistic mechanisms: (1) enhanced surface functionality via magnesium incorporation, (2) improved textural properties from thermal restructuring, and (3) coordinated interactions between mineral phases and carbon matrices [33]. These structural modifications were found to concurrently facilitate chemisorption through active site coordination and physisorption via pore-filling mechanisms. The Langmuir–Freundlich parameter of n > 0.5 confirmed strong adsorbent–adsorbate interactions, reflecting the material’s optimized surface energetics. This dual-mode adsorption mechanism enables efficient nutrient recovery from aqueous systems, with the modified biochar exhibiting 1.9–2.3-fold greater adsorption capacity than conventional counterparts. The demonstrated performance suggests significant potential for application in advanced wastewater treatment systems and closed-loop nutrient recycling processes, particularly for addressing eutrophication challenges while enabling resource recovery.

**Figure 8:**
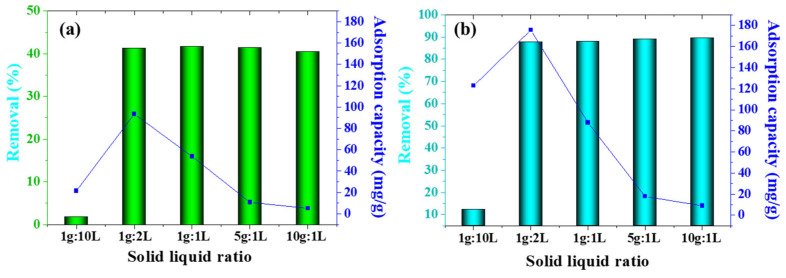
Effect of solid–liquid ratio on nitrogen (**a**) and phosphorus (**b**) removal by Mg-MBC600.

The adsorbent dosage was identified as a critical parameter influencing both adsorption capacity and contaminant removal efficiency in nutrient recovery processes. Experiments were conducted using a simulated aquaculture wastewater solution containing 100 mg/L nitrogen and phosphorus. As illustrated in Figure 8, increasing the dosage of magnesium-loaded co-pyrolysis biochar (Mg-MBC600) from 0.1 to 0.5 g/L enhanced ammonia nitrogen (NH_4_^+^) removal efficiency from 2.45% to 42.71% and total phosphorus (TP) removal efficiency from 13.43% to 86.32%. A further dosage increased to 10 g/L resulted in a marginal decline in NH_4_^+^ removal (40.21%) but elevated TP removal to 88.71%. This behavior suggests that higher adsorbent quantities provide additional active sites for pollutant binding, though saturation effects may limit site utilization efficiency at excessive dosages. Concurrently, the equilibrium adsorption capacity (q_e_) exhibited an inverse relationship with dosage. At 0.5 g/L, Mg-MBC600 demonstrated maximal adsorption capacities of 80.62 mg/g for NH_4_^+^ and 171.35 mg/g for TP. However, these values decreased to 8.07 mg/g (NH_4_^+^) and 10.17 mg/g (TP) at 10 g/L, attributed to incomplete utilization of adsorption sites and reduced mass transfer efficiency under high solid-to-liquid ratios. Based on removal performance and cost-effectiveness, the optimal dosage was determined as 0.5 g/L for subsequent studies.

**Figure 9:**
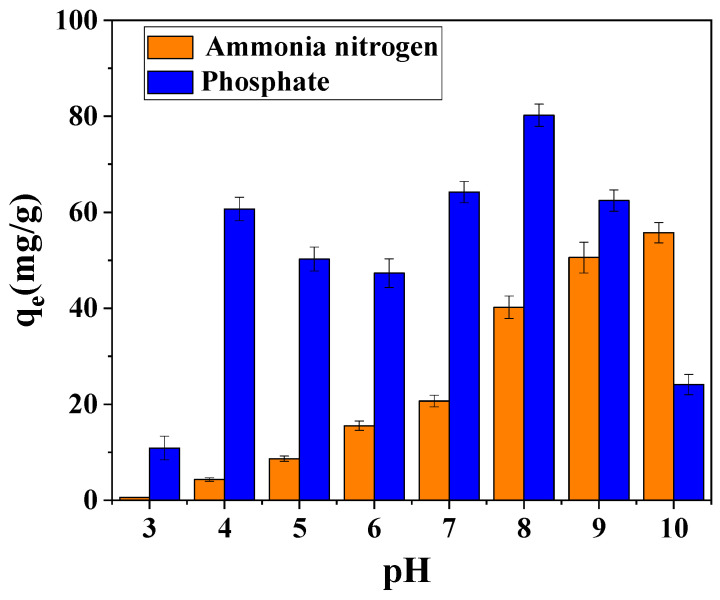
Effect of pH on the adsorption capacity of magnesium-loaded co-pyrolysis biochar.

Solution pH significantly modulated NH_4_^+^ adsorption behavior, as shown in Figure 9. Minimal adsorption (<5%) occurred at pH 2 due to surface protonation of oxygen-containing functional groups (C=O, COO−), which generated positive charges on Mg-MBC600, inducing electrostatic repulsion with NH_4_^+^ cations. Competitive occupation of active sites by H^+^ ions further suppressed adsorption in acidic conditions. Adsorption capacity increased sharply to 40.22 mg/g at pH 8, aligning with the literature reports of enhanced NH_4_^+^ removal in neutral-to-alkaline environments [34]. This improvement was linked to deprotonation of surface groups, creating negatively charged sites favorable for NH_4_^+^ electrostatic attraction. At pH ≥ 10, apparent NH_4_^+^ removal efficiencies exceeding 90% were observed; however, this phenomenon was attributed to NH_3_ volatilization rather than true adsorption, as confirmed by mass balance analyses [35].

The pH-dependent adsorption behavior of total phosphorus (TP) on magnesium-modified co-pyrolysis biochar (Mg-MBC600) is illustrated in Figure 9. At pH 2, negative sorption capacities (originally presented at pH 2) reflect net desorption/leaching of inherent phosphorus from the biochar matrix, now displayed as zero, with “desorption” denoted in parentheses. Conversely, at pH 4, near-complete suppression of leaching (<8% P release) enabled adsorption efficiency of 60.69 mg/g TP, as surface charge neutralization stabilized mineral-bound P while promoting ligand exchange with Mg sites.

The optimal phosphorus removal capacity of 80.21 mg/g TP at pH 8 was achieved through three synergistic mechanisms: (1) electrostatic attraction between deprotonated surface functional groups and anionic phosphate species (HPO_4_^2−^/PO_4_^3−^), (2) ligand exchange reactions mediated by Mg^2+^ immobilized on the carbon matrix, and (3) reduced competitive occupation of adsorption sites by H^+^. A marked reduction in TP adsorption (≤55 mg/g) at pH ≥ 10 was attributed to hydroxyl ion competition and the formation of soluble Mg(OH)_2_ complexes that sequestered available Mg^2+^ for phosphate binding. Enhanced nutrient removal in the neutral pH range (7–8) was further governed by magnesium ammonium phosphate crystallization (struvite, MgNH_4_PO_4_·6H_2_O), where surface-exposed Mg^2+^ sites demonstrated three critical functions: (a) electrostatic concentration of NH_4_^+^ and PO_4_^3−^, (b) nucleation promotion for struvite precipitation, and (c) maintenance of localized alkaline microenvironments (pH 8.5–9.5) through the biochar’s intrinsic buffering capacity, despite neutral bulk solution conditions. This dual adsorption–precipitation mechanism was structurally confirmed via SEM-EDS analysis, which identified orthorhombic struvite crystals (10–25 μm) preferentially formed at Mg-rich surface regions and pore openings. The pH-dependent speciation of magnesium (Mg^2+^→Mg(OH)^+^→Mg(OH)_2_) was identified as the principal regulator of both adsorption dynamics and crystallization processes, demonstrating the critical role of coordination chemistry in governing the material’s phosphorus sequestration performance.

**Figure 10:**
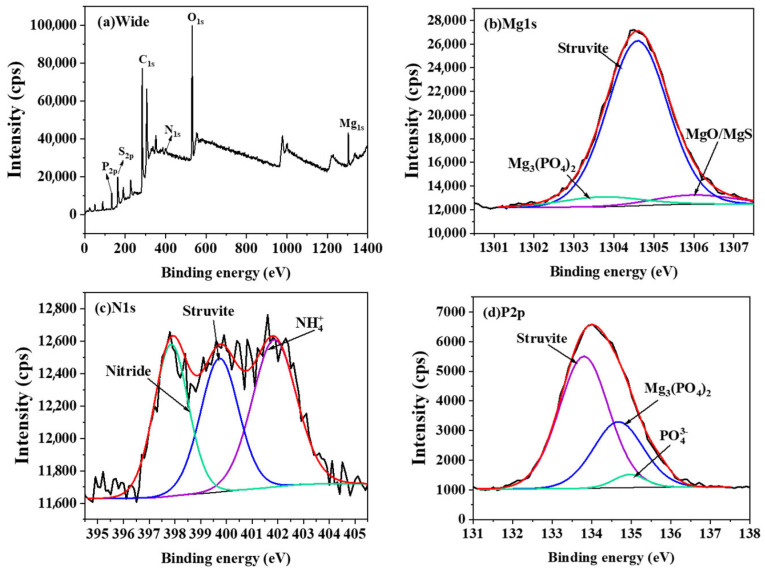
XPS spectra of magnesium-loaded co-pyrolysis modified biochar after simultaneous adsorption of nitrogen and phosphorus (full spectrum (**a**), Mg 1s (**b**), N 1s (**c**), and P 2p (**d**)).

The surface composition and adsorption mechanisms of magnesium-loaded co-pyrolysis biochar (Mg-MBC600) were systematically investigated through X-ray photoelectron spectroscopy (XPS). As shown in Figure 10a, the full spectrum confirmed the presence of Mg, N, P, S, and O, verifying successful magnesium incorporation and nutrient retention, in which the black and red line mean the spectrum and fitted curve. Deconvolution of the Mg1s spectrum (Figure 10b) revealed three distinct binding energy components: 1306.0 eV (attributed to MgO/MgS species), 1303.5 eV (assigned to Mg^2+^ in magnesium phosphate complexes), and 1304.6 eV (dominant peak corresponding to struvite crystallization products). Post-adsorption analysis of the N1s spectrum (Figure 10c) identified three nitrogen configurations: a primary peak at 400.2 eV (N-P coordination bonds in phosphate associations), a secondary component at 402.0 eV (NH_4_^+^ adsorbed through electrostatic interactions or cation exchange with Na^+^/Mg^2+^), and a tertiary signal at 398.5 eV (structural N-C/N=C bonding within the carbon matrix). The P 2p spectrum (Figure 10d) exhibited three characteristic peaks at 133.8 eV (Mg(NH_4_)PO_4_), 135.0 eV (Mg_3_(PO_4_)_2_), and 135.2 eV (adsorbed PO_4_^3−^), enabling quantitative differentiation between surface adsorption (10% contribution) and co-precipitation (90% dominance) mechanisms [36]. Semi-quantitative analysis based on peak area integration demonstrated that phosphorus removal predominantly occurred through magnesium-mediated precipitation processes, with struvite (MgNH_4_PO_4_·6H_2_O) and magnesium phosphate compounds accounting for 87.3% of total sequestration. Only 12.7% of phosphorus removal was attributed to direct surface adsorption, confirming the critical role of pH-dependent magnesium speciation in governing nutrient immobilization pathways. These findings corroborate established mechanisms wherein magnesium-modified biochars enhance phosphate removal (>90% efficiency) via synergistic chemisorption—predominantly ligand exchange at Mg-OH sites and struvite precipitation.

A comparison of this work with previous biochar for N and P adsorption was conducted (in Table 5), and it revealed that Mg-MBC600 has a higher adsorption capacity for N and P than almost-modified biochar, which further demonstrated that Mg-modified biochar derived from co-pyrolysis for sludge and biomass can be used as a potential adsorbent for N and P from sewage. The conversion of “wasted” sludge and biomass into a “useful” biochar for N and P adsorption meets the requirement of a circular economy.

## 4. Conclusions

This study systematically investigated the modification and performance mechanisms of magnesium-loaded biochar derived from co-pyrolysis of sludge and biomass for simultaneous nitrogen and phosphorus removal from wastewater. The magnesium-impregnated composite biochar exhibited enhanced textural characteristics, achieving a specific surface area of 156.08 m^2^/g and pore volume of 0.1829 cm^3^/g through optimized co-pyrolysis conditions with agricultural straw. Structural characterization revealed the successful formation of magnesium oxide crystalline phases, with nano-sized MgO particles (approximately 50 nm diameter) uniformly distributed across the carbon matrix, as confirmed by advanced microscopic analysis. Adsorption kinetic studies demonstrated superior correlation with the pseudo-second-order model (R^2^ > 0.99), indicating chemisorption-dominated removal mechanisms for both nitrogen and phosphorus species. Equilibrium isotherm analysis suggested a hybrid adsorption behavior, combining monolayer coverage and multilayer heterogeneous adsorption processes. The maximum adsorption capacities were quantified as 47.17 mg/g for ammonia nitrogen and 91.09 mg/g for total phosphorus, representing significant enhancement compared to conventional sludge-derived biochars. This performance improvement was attributed to two synergistic factors: increased specific surface area and mesoporous structure facilitating physical adsorption, and magnesium-mediated chemical interactions with target pollutants. Solution pH was identified as a critical operational parameter. X-ray photoelectron spectroscopy analysis confirmed the predominant role of precipitation mechanisms, particularly through magnesium–phosphate crystallization and struvite-like compound formation. The combined physicochemical characterization and adsorption experiments established that the modified biochar achieves concurrent nutrient removal through multiple pathways, including surface complexation, electrostatic attraction, and chemical precipitation. Future investigations will quantitatively characterize the slow-release behavior of spent biochar through standardized soil-column leaching with kinetic modeling, while encompassing comprehensive risk assessment via BCR sequential extraction of heavy metals, ecotoxicological bioassays, and expanded techno-economic analysis comparing lifecycle costs against commercial alternatives.

## Figures and Tables

**Table 1:** Analysis of basic physical and chemical properties of raw materials.

Sample	Proximate Analysis/%				Ultimate Analysis/%						BET (m^2^/g)
	M_ad_	A_ad_	V_ad_	FC_ad_	C_ad_	H_ad_	N_ad_	O_ad_ *	S_ad_	H/C_ad_	
Sunflower Stalk	5.81	3.55	84.4	6.24	47.04	5.02	0.29	31.72	0.33	1.28	26.87
Sludge	5.21	63.74	35.07	1.19	15.44	3.25	1.62	14.14	0.55	2.53	17.47

Note: M_ad_ means moisture content, A_ad_ means ash content, and V_ad_ means volatiles * By difference, O_ad_ = 100 − (C + H + N + S + ash).

**Table 2:** Structural characteristics and elemental composition analysis of magnesium-loaded biochar.

Sample Name	Elemental Composition (wt%)				Specific Surface Area (m^2^/g)	Pore Volume (cm^3^/g)	Average Pore Size (nm)
	C	H	N	Mg			
Sewage peat	10.63	1.26	0.72	1.32	49.62	0.1685	14.87
Co-pyrolysis carbon	46.57	1.36	1.72	0.87	124.36	0.1764	4.23
Magnesium-loaded modified biochar	43.26	1.32	1.66	9.86	156.08	0.1829	3.67

**Table 3:** Kinetic parameters of nitrogen and phosphorus adsorption on magnesium-modified biochar.

Sample	Adsorbate	Pseudo-First Order			Pseudo-Second Order			Elovich		
		k_1_	q_e_	R^2^	k_2_	q_e_	R^2^	α	β	R^2^
Mg-MBC	NH_4_^+^	0.0756	22.55	0.9469	0.005	23.84	0.9901	363.08	0.4698	0.7956
Mg-MBC	TP	0.0114	81.91	0.9224	0.001	95.45	0.9844	3.1758	0.05	0.9716

**Table 4:** Nitrogen and phosphorus adsorption isotherm parameters of magnesium-modified biochar.

Sample Name	Adsorbate	Langmuir			Freundlich			Langmuir–Freundlich			
		K_L_	q_m_	R^2^	K_F_	n	R^2^	k	n	q_m_	R^2^
Mg-MBC600	Ammonia nitrogen	0.1048	52.56	0.9359	17.7376	4.5171	0.9133	0.0452	0.6496	47.17	0.9567
	Total phosphorus	0.006	110.29	0.9845	6.3103	2.3826	0.9703	0.001	0.6987	91.09	0.9971

**Table 5:** Comparison of this study with relevant recent studies of modified biochar for N and P adsorptive removal.

Precursor	Modification Agent	pH	q_e_ (mg NH_4_^+^/g)	q_e_ (mg TP/g)	Kinetic Model	Adsorbent Model	Ref.
Corn stalk	Magnesium	9–11	177.25	253.95 (PO_4_^3−^)	Pseudo-second order	Langmuir–Freundlich	[5]
Macrophyte cattails and sludge	HDPE	10	111.96	107.72 (PO_4_^3−^)	Pseudo-second order	Langmuir	[34]
Peanut shell and purified bentonite	MgCl_2_ CaCl_2_	7	39.5	132.2 (PO_4_^3−^)	Pseudo-second order	Langmuir–Freundlich	[37]
Corn cob	AlCl_3_·6H_2_O	5–6	-	44.79 (PO_4_^3−^)	Pseudo-second order	Langmuir	[38]
Zeolite powders	NaCl	6–7	12.0	9.3 (PO_4_^3−^)	Pseudo-second order	Langmuir	[39]
Coal gasification slag	NaOH	6–8	7.44	6.94 (PO_4_^3−^)	Pseudo-second order	Langmuir	[40]
Oak wood and greenhouse waste; anaerobically waste	Ethanol ammonium acetate	6–9	146.4	30 (PO_4_^3−^)	Pseudo-second order	Langmuir–Freundlich	[41]
Municipal waste	MgCl_2_	7–9	32.01	109.58 (PO_4_^3−^)	Pseudo-second order	Langmuir–Freundlich	[42]
Municipal sewage sludge and walnut shell	Material ratio	7–9	22.85	303.49 (PO_4_^3−^)	Pseudo-second order	Langmuir–Freundlich	[43]
Sewage sludge fly ash and clay ceramsite	NaOH/NaCl LaCl_3_/NaOH	6–8	12.52	0.93 (PO_4_^3−^)	Pseudo-second order	Langmuir	[18]
Bamboo powder and montmorillonite		7	12.52	105.28 (PO_4_^3−^)	Pseudo-first order	Langmuir	[44]
Wheat straw, apple branches and kiwi branches	HCl FeCl_3_	7	22.98	28.10 (PO_4_^3−^)	Pseudo-second order	Langmuir	[45]
Sludge and sunflower stalk	MgO	3–9	84.92	182.27	Pseudo-second order	Langmuir–Freundlich	This work

## Data Availability

The datasets used or analyzed during the current study are available from the corresponding author upon reasonable request.
